# Predictors of Failed Spinal Arachnoid Puncture Procedures: An Artificial Neural Network Analysis

**DOI:** 10.7759/cureus.32891

**Published:** 2022-12-23

**Authors:** Habib Md R Karim

**Affiliations:** 1 Anesthesia, Critical Care, and Pain Medicine, All India Institute of Medical Sciences, Raipur, IND

**Keywords:** neuraxial anesthesia, lumber puncture, failure, spinal column, procedures, machine learning

## Abstract

Background

Knowing the predicting factors for difficult neuraxial blocks might help better plan the procedure. This study aimed to determine the predictors of failed spinal arachnoid puncture procedures using artificial neural network (ANN) analysis.

Methodology

With approvals, prospectively collected data from 300 spinal arachnoid punctures in the operation theater of an academic institute having postgraduate anesthesia training were retrospectively evaluated. Fifteen variables from anthropo-demographic, spinal surface anatomy, procedure, and performers' experiences were fed as input for the ANN. A failed spinal arachnoid puncture procedure was defined as the requirement of more than three punctures, with three punctures but more than six passes, or if the performer handed over the procedure to another, considering it difficult after the second puncture. STATCRAFT v.2 software (Predictive Analytics Solutions Pvt. Ltd., Bengaluru, India) was used for ANN model generation. Considering the overfitting tendency of the ANN, Pr(>|*z*|) < 0.01 in the ANN was considered significant. The area under the receiver operating characteristic (AuROC) curve of the ANN model and its sensitivity and specificity were also assessed. Significant factors with multiple gradings were also evaluated for their statistical significance across the grades or classes using INSTAT software (Graphpad Prism, La Jolla, CA, USA); a two-tailed *P*-value of <0.05 was considered significant.

Results

Interspinous process-based spine grade, performers' experience, and positioning difficulty were significant determinants of failed spinal arachnoid puncture procedures in the ANN model. The ANN model had an AuROC of 0.907, specificity of 0.976, and sensitivity of 0.385. The interclass comparison showed that increasing spinal grades and decreasing experiences were associated with increased pass and puncture.

Conclusions

The ANN model found the determinants of the failed spinal arachnoid puncture procedure well with good AuROC and specificity but poor sensitivity.

## Introduction

Spinal anesthesia and spinal (lumber) puncture are standard procedures performed in healthcare practice. Although the technique appears to be sufficiently straightforward, substantial skill is required for a successful arachnoid puncture, especially for first-pass success and atraumatic puncture. Repeated encounters with the surrounding osseous structure can make the spinal arachnoid space challenging to access, leading to trauma and procedural failure; correct identification of the puncture point and optimal angulation of the needle trajectory path are crucial. While most of the difficulties and failures might be technical, patients' back anatomy, the performer's experience, or other procedural aspects might impact it. The difficulty bears importance from the procedural outcome and the patient's discomfort arising from the need for multiple punctures. On the other hand, it might have an acute and long-term effect on the patient’s health [1,2]. Further, such patients are likely to refuse subsequent neuraxial anesthesia even if there is an outweighing benefit [3].

Deep machine learning is gaining popularity in helping identify and assist clinicians in different aspects; finding out the factors responsible for a cause (clinical problem) is also one such area. Therefore, the following research question was postulated *Can deep machine learning identify the factors associated with a difficult spinal arachnoid puncture in patients undergoing spinal arachnoid block in the operating room?* And this research was planned. The study aimed to identify the factors associated with a failed spinal arachnoid puncture using artificial neural network (ANN) analysis.

## Materials and methods

This study proposal was approved by the Institute Research Cell (Project Code No. AIIMS-RPR/IRC/IM/NF/2022/433). The Institute Ethics Committee approved it with a consent waiver (No. 2484/IEC-AIIMSRPR/2022). As the data were analyzed retrospectively, a clinical trial registry was not done for the study.

This study was conducted in an academic institute having postgraduate and postdoctoral academic training in anesthesiology in India. Although the study involves retrospective data analysis, all of these data were prospectively observed and collected from the anesthesia management and patient file. Prior information for the data bank creation for future use was given to the head of the department by the project's principal investigator, and data were stored in Excel format. Data were from the patients undergoing spinal anesthesia in our operation theaters; adults undergoing any surgery under spinal arachnoid block performed in sitting and lateral positions were included. The data collection process did not affect the decision to perform the subarachnoid block (SAB), indication, contraindication of the SAB, and any procedure-related plan and management. Patients undergoing combined spinal anesthesia, where a separate spinal puncture is done after epidural; pregnant women; and patients undergoing spinal anesthesia in the prone position were excluded. The sample size of this study was calculated based on the incidence of difficult spinal anesthesia found by Atallah et al. [4], i.e., 12.7%. We calculated the sample for an infinite population with an absolute error of 5%, which gave a size of 295 for a 99% confidence interval. Clinicodemographic data of the patient, experience of the performer, numbers of passes and punctures, and thoraco-lumbosacral spine surface anatomy-related data were collected. Further data on positioning difficulty, the position at which the puncture is attempted, and performers' experience in months of anesthesia training were also collected. Data were segregated and categorized as per the predefined classification adopted for this study. Further, the procedure was classified as a success or failure using the definition adopted for this study.

A study that analyzed the anatomical factors that reduced the ability to bend the waist and the inability to palpate the spinous process and space had the predicting ability for difficult spinal anesthesia [2]. The practice of inspection and palpation (superficial and deep) was incorporated for clinical examination. Both the vertical and horizontal anatomical planes were considered for the classifications and gradings on the surface anatomy. The following classes or grades were used in this study:

-*Spine grades (based on interspinous space in the lumber area)*: grade 1 - spinous processes and intervertebral space can be guessed by inspection; grade 2 - spinous processes and intervertebral space can be felt by superficial palpation; grade 3 - spinous processes and intervertebral space can be felt by deep (with pressure) palpation; and grade 4 - spinous processes and intervertebral space cannot be felt even by deep palpation.

-*Thoraco-lumbosacral spine contour with a request to flex (for the longitudinal plane)*: grade 1 - convex; grade 2 - straight (flat/plain); and grade 3 - concave.

-*Spinous process and bilateral paravertebral muscles couture (for the horizontal plane):* grade 1 - convex; grade 2 - straight (flat/plain); and grade 3 - concave (scaphoid).

Further, we also considered experience as one of the factors. We graded the experience as grade 1, residents with less than 6 months experience in academic anesthesia training; grade 2, residents with 6 to 12 months of experience in academic anesthesia training; grade 3, residents with 12 to 24 months of experience in academic anesthesia training; and grade 4, experienced (qualified) anesthesiologists.

Be noted that in India, a diploma in anesthesia is a recognized qualification for anesthesiologists, which has 24 months of academic training after the Bachelor of Medicine and Bachelor of Surgery. Therefore, the study considered an academic resident who has completed 24 months of training during Doctor of Medicine (Anesthesiology) as experienced, i.e., grade 4 experience. 

A robust, universally accepted definition for failed, difficult, and easy spinal arachnoid puncture procedure was not found in the literature. For the development of difficulty scores, Atallah et al. used pass and puncture as guidance for defining difficulty [4]. In this study, these were considered composite outcomes for defining the criteria. The spinal arachnoid puncture procedure was categorized as failed if a performer required more than three punctures or with three punctures but more than six passes or if the performer handed over the procedure to another performer, considering it difficult after the second puncture. Puncture for this purpose was taken as the needle puncturing the skin surface and entering the patient. Passes were defined as simply redirecting the needle without removing the needle from the patient.

The prospectively collected data bank was stored in Excel format; the same was used as the master chart for the present retrospective evaluation. If any set of demographic data for a particular patient was missing, the mean value of the entire cohort was used for that missing set. The cohort was then subgrouped based on success or failure as per the definition used for this study. Categorical variables were presented as absolute numbers and percentage scales. Continuous data were presented as mean ± SD. The data distribution was assessed using the Kolmogorov-Smirnov test, and groups were compared using the unpaired t-test or Mann-Whitney test based on the data distribution. The 2 × 2 contingency table data were compared using Fisher’s exact test, and the chi-square test for independence was used for larger contingency tables. A two-tailed *P*-value <0.05 was considered statistically significant for univariate analysis. INSTAT software (Graphpad Prism, La Jolla, CA, United States) was used for statistics. The ANN analysis was done using a deep machine-learning platform from STATCRAFT (version 2.0, a browser-based software powered by R from Predictive Analytics Solutions Pvt. Ltd., Bengaluru, India). The software inherently sets the dataset division into training and testing. Considering the susceptibility of overfitting due to the increased flexibility of ANN, the Pr(>|*z*|) value of <0.01 was considered significant for the model [5]. The study results and reporting follow the Strengthening the Reporting of Observational Studies in Epidemiology guideline [6].

## Results

Data from 297 patients undergoing 300 spinal anesthesia procedures from July 2018 to June 2019 were included in the study. The mean ± SD of the age of the cohort was 48.73 ± 18.16 years, height 161.80 ± 9.30 cm, weight 62.29 ± 14.84 kg, and BMI 23.76 ± 5.18 km/m^2^ of the body surface area. The American Society of Anesthesiologists Physical Class (ASA-PS) distribution of the cohort was 25.33%, 56.33%, 16.33%, and 2.0%, respectively, for classes I, II, III, and IV. Only 35 (11.66%) of the cohort were obese. Two patients had no data on the weight and height where mean values of the cohort were used. For the statistical analysis purpose, the gender of the patient was counted as the number of the spinal arachnoid puncture procedure; if the same patient underwent spinal arachnoid puncture twice, gender was counted twice, i.e., 300 total; 227 (75.66%) procedures were performed in male.

A total of 15 variables were taken as the input for the ANN analysis to predict the failure as the outcome per the adopted definition. Of the total number of procedures, 39 (13%) were categorized as failed spinal arachnoid puncture procedures according to the adopted definition in this study. The ANN model showed a significant Pr(>|*z*|) value <0.01 only for spinal grades, the experience of the performer, and positioning difficulty as the predictor of the success or failed spinal arachnoid puncture (Table 1).

**Table 1:** Artificial neural network model results and odds of the input variables fed for predicting failed spinal arachnoid puncture procedures. ASA-PS, American Society of Anesthesiologists; BMI, body mass index; LA, local anesthesia; TLS, thoraco-lumbosacral; TPM, transverse paravertebral muscle; SE, standard error; OR, odds ratio

							OR and confidence intervals		
Coefficients	Estimate	SE	2.5% SE	97.5% SE	*z* value	Pr(>|*z*|)	OR	2.5 %	97.5 %
(Intercept)	12.135	22.515	-31.994	56.265	0.539	0.589	186462.754	1.601e^-14^	8.136e^+24^
Age	0.030	0.017	-0.004	0.064	1.726	0.084	1.030	0.996	1.068
Sex	-1.834	0.852	-3.505	-0.164	-2.152	0.031	0.159	0.027	0.801
Weight	0.001	0.121	-0.236	0.239	0.011	0.990	1.001	0.787	1.272
Height	0.0483	0.112	-0.171	0.268	0.431	0.666	1.049	0.841	1.311
BMI	-0.011	0.322	-0.643	0.621	-0.035	0.972	0.988	0.521	1.865
ASA-PS	-0.467	0.392	-1.236	0.301	-1.191	0.233	0.626	0.285	1.343
Spine grades	-2.715	0.537	-3.768	-1.662	-5.055	4.287e-7	0.066	0.021	0.176
TPM contour	-0.740	0.518	-1.756	0.274	-1.429	0.152	0.476	0.166	1.288
TLS contour	0.705	0.553	-0.379	1.790	1.275	0.202	2.025	0.691	6.152
Spine abnormality	-1.380	2.118	-5.533	2.771	-0.651	0.514	0.251	0.006	18.108
Experience	1.045	0.274	0.506	1.584	3.801	0.0001	2.844	1.713	5.075
Patient position	16.249	1157.404	-2252.222	2284.721	0.014	0.988	11400140.315	4.456e^-17^	1.235e^+162^
Position difficulty	-2.980	0.950	-4.844	-1.116	-3.134	0.001	0.050	0.007	0.345
Needle	-0.458	0.575	-1.586	0.669	-0.796	0.425	0.632	0.211	2.063
Preprocedural LA	-0.676	0.630	-1.911	0.559	-1.072	0.283	0.508	0.140	1.699

**Figure 1:**
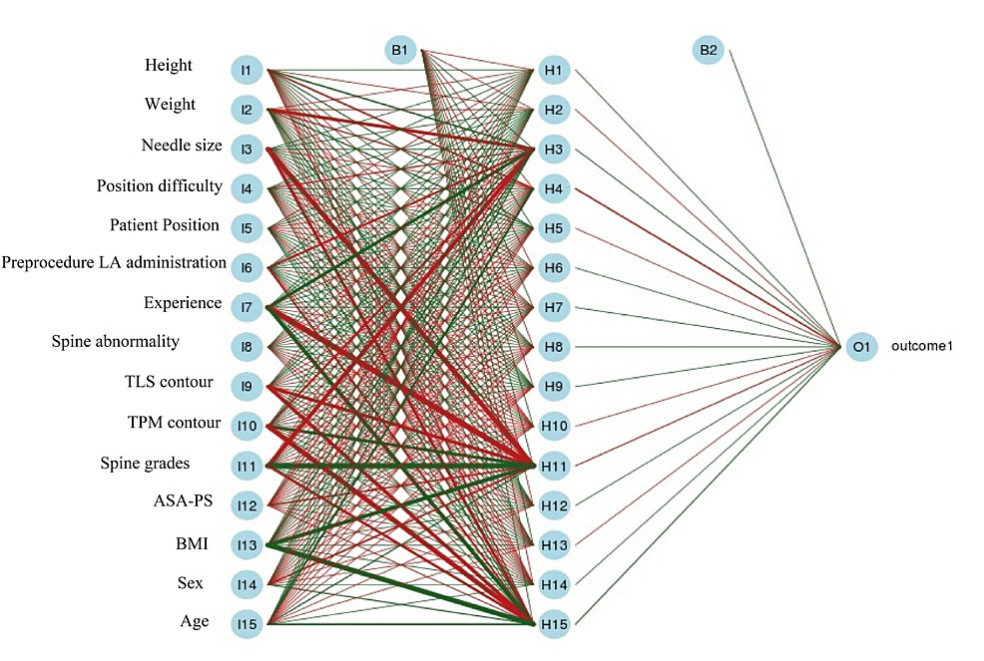
The artificial neural network architecture for failed spinal arachnoid puncture procedures. Note: The input parameters of the architecture are rewritten in Paint for clarity. ASA-PS, American Society of Anesthesiologists; BMI, body mass index; LA, local anesthesia; TLS, thoraco-lumbosacral, TPM, transverse paravertebral muscle

**Figure 2:**
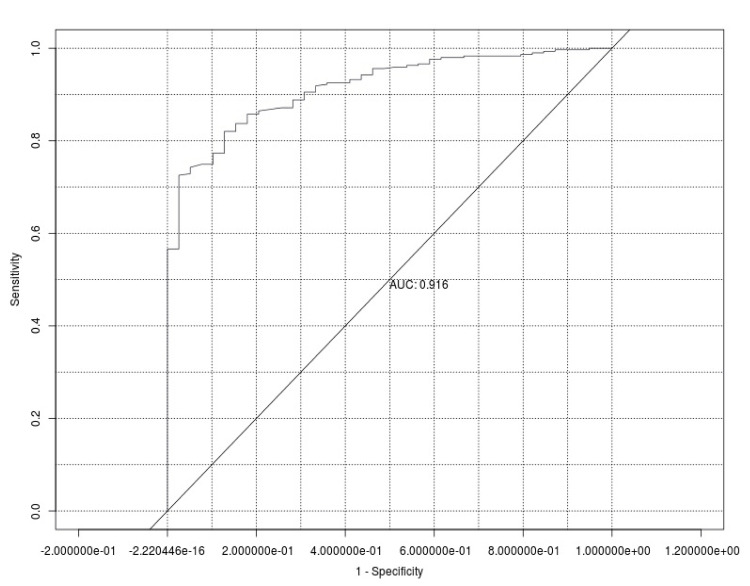
The receiver operating characteristics and area under the artificial neural network model curve for predicting failed spinal arachnoid puncture procedures. AUC, area under the curve

The overall statistical result of the ANN showed an accuracy of 0.907 (0.870-0.936) and a Mcnemar *P*-value of 0.004. The ANN architecture is presented in Figure 1. The ANN model showed an excellent area under the ROC curve (AuROC), i.e., 0.916 (Figure 2).

Interclass analysis of the different spine grades used in the study as per the surface anatomy findings on inspection and palpation for their association with the number of passes and punctures required also showed that spine grade 1 required a significantly lower number of punctures and had better first puncture success than spine grades 2, 3, and 4 (*P* = 0.0005, <0.0001, and <0.0001, respectively). The same was true for the number of passes and first-pass success rate (Table 2).

**Table 2:** Comparison showing the relationship of different spinal grades with the anthropo-demographic and performance parameters. ASA-PS, American Society of Anesthesiologists Physical Class

Parameters	Spine grade 1 (*N* = 95)	Spine grade 2 (*N* = 148)	*P*-value	Spine grade 3 (*N* = 52)	*P*-value	Spine grade 4 (*N* = 05)	*P*-value
Age (years)	41.85 ± 17.27	44.72 ± 16.04	0.187	46.87 ± 16.20	0.022	54.58 ± 19.05	0.107
Male	74 (77.89)	118 (79.72)	0.748	31 (59.61)	0.022	04 (80.0)	1.000
Female	21 (22.10)	30 (20.27)	0.748	21 (40.38)	0.022	01 (20.0)	1.000
Height (cm)	161.98 ± 9.04	162.28 ± 8.61	0.797	160.98 ± 9.84	0.427	166.6 ± 16.08	0.289
Weight (kg)	53.87 ± 10.40	62.08 ± 11.71	<0.0001	67.04 ± 13.05	<0.0001	102 ± 38.38	<0.0001
Body mass index	20.46 ± 3.31	23.49 ± 3.60	<0.0001	25.90 ± 4.90	<0.0001	35.59 ± 9.51	<0.0001
ASA-PS: I	31 (32.63)	40 (27.02)	0.386	05 (9.61)	0.002	0	0.320
ASA-PS: II	55 (57.89)	88 (59.45)	0.893	23 (44.23)	0.123	03 (60.0)	1.000
ASA-PS: III	09 (9.47)	18 (12.16)	0.676	21 (40.38)	<0.0001	01 (20.0)	0.416
ASA-PS: IV	0	02 (1.35)	0.521	03 (5.76)	0.042	01 (20.0)	0.050
No. of puncture: 1	88 (92.63)	111 (75.0)	0.0005	20 (38.46)	<0.0001	00	<0.0001
No. of puncture: 2	06 (6.31)	31 (20.94)	0.001	23 (44.23)	<0.0001	03 (60.0)	0.004
No. of puncture: 3	01 (1.05)	05 (3.37)	0.408	08 (15.38)	0.001	02 (40.0)	0.005
No. of puncture: 4	00	01 (0.67)	1.000	01 (1.92)	0.353	00	-
No. of passes: 1	68 (71.57)	73 (49.32)	0.0008	08 (15.38)	<0.0001	00	0.002
No. of passes: 2	16 (16.84)	28 (18.91)	0.735	10 (19.23)	0.821	00	1.000
No. of passes: 3	06 (6.31)	16 (10.81)	0.261	04 (7.69)	0.742	00	1.000
No. of passes: 4	03 (3.15)	09 (6.08)	0.375	05 (9.61)	0.131	01 (20.0)	0.188
No. of passes: 5	02 (2.10)	09 (6.08)	0.209	06 (11.53)	0.023	00	1.000
No. of passes: 6	00	10 (6.75)	0.007	08 (15.38)	0.0002	02 (40.0)	0.002
No. of passes: >6	00	03 (2.02)	0.282	11 (21.15)	<0.0001	02 (40.0)	0.002
Spine deformity: Yes	03 (3.15)	02 (1.35)	0.382	01 (1.92)	1.000	01 (20.0)	0.188
Spine deformity: No	92 (96.84)	146 (98.64)	0.382	51 (98.07)	1.000	04 (80.0)	0.188
Position: Lateral	02 (2.11)	04 (2.70)	1.000	00	0.539	00	1.000
Position: Sitting	93 (97.89)	144 (97.29)	1.000	52 (100)	0.539	05 (100)	1.000
Position difficulty: Yes	01 (1.05)	08 (5.40)	0.094	02 (3.84)	0.285	00	1.000
Position difficulty: No	94 (98.94)	140 (94.59)	0.094	50 (96.15)	0.285	05 (100)	1.000

However, analysis of the different levels of experiences categorized in the study as per the training duration for their association with the number of passes and punctures showed no difference in the first puncture success. Nevertheless, it showed that experience classes 3 and 4 had better chances of a successful spinal puncture on the first pass (Table 3), even if the patients had higher spine grades and had spinal abnormalities.

**Table 3:** Comparison showing the relation of different levels of performers' experiences with the anthropo-demographic and performance parameters. ASA-PS, American Society of Anesthesiologists Physical Class; Exp., experience

Parameters	Exp. class 1 (*N* = 114)	Exp. class 2 (*N* = 82)	*P*-value	Exp. class 3 (*N* = 33)	*P*-value	Exp. class 4 (*N* = 71)	*P*-value
Age (years)	45.81 ± 15.30	41.71 ± 15.74	0.069	43.07 ± 17.21	0.243	47.61 ± 18.32	0.456
Male	80 (70.17)	71 (86.58)	0.009	28 (84.84)	0.118	48 (67.60)	0.745
Female	34 (29.82)	11 (13.41)	0.009	05 (15.15)	0.118	23 (32.39)	0.745
Height (cm)	160.66 ± 8.65	163.90 ± 8.89	0.011	163.45 ± 9.41	0.035	162.1 ± 10.43	0.298
Weight (kg)	59.97 ± 11.49	61.72 ± 17.34	0.396	60.17 ± 17.38	0.921	62.52 ± 20.57	0.271
Body mass index	23.25 ± 4.39	23.53 ± 4.64	0.674	23.29 ± 4.55	0.950	24.75 ± 6.67	0.060
ASA-PS: I	31 (27.19)	18 (21.95)	0.503	08 (24.24)	0.825	19 (26.76)	1.000
ASA-PS: II	65 (57.01)	50 (60.97)	0.659	21 (63.63)	0.551	33 (46.47)	0.175
ASA-PS: III	17 (14.91)	12 (14.63)	1.000	04 (12.12)	0.785	16 (22.53)	0.236
ASA-PS: IV	01 (0.87)	02 (2.43)	0.572	0	1.000	03 (4.22)	0.158
No. of punctures: 1	82 (71.92)	55 (67.07)	0.528	25 (75.75)	0.824	57 (80.28)	0.224
No. of punctures: 2	28 (24.56)	20 (24.39)	1.000	06 (18.18)	0.493	09 (12.67)	0.059
No. of punctures: 3	04 (3.50)	06 (7.31)	0.325	01 (3.03)	1.000	05 (7.04)	0.307
No. of punctures: 4	0	01 (1.21)	0.418	01 (3.03)	0.224	0	-
No. of passes: 1	50 (43.85)	39 (47.56)	0.663	21 (63.63)	0.050	39 (54.92)	0.173
No. of passes: 2	22 (19.29)	14 (17.07)	00713	03 (9.09)	0.199	15 (21.12)	0.850
No. of passes: 3	10 (8.77)	08 (9.75)	0.807	02 (6.06)	1.000	06 (8.45)	1.000
No. of passes: 4	08 (7.01)	03 (3.65)	0.364	05 (6.09)	0.167	02 (2.81)	0.321
No. of passes: 5	06 (5.26)	09 (10.97)	0.175	01 (3.03)	1.000	01 (1.40)	0.253
No. of passes: 6	13 (11.40)	04 (4.87)	0.128	00	0.041	03 (4.22)	0.111
No. of passes: >6	05 (4.38)	05 (5.7)	0.744	01 (3.03)	1.000	05 (7.3)	0.510
Spine abnormality: Yes	01 (0.87)	0	1.000	0	1.000	06 (8.45)	0.013
Spine abnormality: No	113 (99.12)	82 (100)	1.000	33 (100)	1.000	65 (91.54)	0.013
Position: Lateral	0	01 (1.21)	0.418	0	-	05 (7.05)	0.007
Position: Sitting	114 (100)	81 (98.78)	0.418	33 (100)	-	66 (92.95)	0.007
Position difficulty: Yes	01 (0.87)	02 (2.43)	0.572	02 (6.06)	0.126	06 (8.45)	0.013
Position difficulty: No	113 (99.12)	80 (97.56)	0.572	31 (93.93)	0.126	65 (91.54)	0.013

## Discussion

The present deep machine-learning-based analysis of patient characteristics, surface landmark-based spinal anatomy, procedure-related factors, and performers' experience showed excellent predictability of failed spinal arachnoid puncture procedures with an AuROC curve of 0.907. Although the model had an excellent specificity of 0.976, the sensitivity was low, i.e., 0.385. Out of the 15 factors analyzed, only spine grade was a significant predictor in the univariate and ANN models. The ANN model also showed the experience of the performer and position difficulty as significant, which was not found in the univariate analysis. It might be explained by the fact that the spinal arachnoid puncture procedure in the cases with spinal deformity and positioning difficulty were performed by the performers with higher experiences. As the experienced person had lower chances of failure, it resulted in higher success, and the difference in the univariate analysis was masked. The same also explains the finding of positioning difficulty as a significant predictor in the ANN model. Further, the presence of positioning difficulty as a parameter was subjective and subject to bias in this study, unlike other parameters that were objective or quantified by adopting a definition.

In a study including 300 Egyptian patients undergoing urological surgeries performed under spinal anesthesia, Atallah et al. found that the first-pass success was 87.3%; 12.7% of cases required a second or more puncture, even more than one space puncture [4]. Our study showed almost similar numbers of failed spinal arachnoid puncture procedures. The success rate on the first attempt was only 61.51% in a study by de Filho GR et al., in which the authors included 1,481 Brazilian patients undergoing either spinal anesthesia or epidural anesthesia [7]. Prakash et al.’s prospective, observational study, including 1,641 patients undergoing spinal anesthesia in India, found that 47.1% of patients required more than one skin puncture attempt to have a successful spinal arachnoid puncture [8]. A Croatian study including 316 patients also found the incidence of multiple puncture requirements in 30.7% of neuraxial procedures [9].

Multiple studies have been carried out to find the predictors or risk factors for difficult or failed spinal arachnoid punctures. These studies have primarily addressed the anatomical and demographic parameters of the patients and the performer's experience using a univariate and logistic regression model. An Iranian study, including data from 109 pregnant women undergoing elective cesarean delivery under spinal anesthesia, assessed the surface anatomical characteristics of the spine concerning the difficult spinal anesthesia [2]. The investigators found that increasing age, weight, BMI, poor identification, or inability to identify the interspinous space were predictors of difficulty. These findings echo the finding of Ružman et al. [9]. Further, Ružman et al. also found a recumbent position, and deFilho et al. found the performer's experience to predict difficult spinal arachnoid puncture [7,9].

Results of the present ANN analysis for finding the factors associated with failed spinal arachnoid puncture showed odds of more than 1 for weight and height. BMI is derived from weight and height and might be a determinant. Although our ANN model did not find BMI as a significant predictor, the failed group had a significantly higher BMI (27.3 ± 5.9 versus 23.2 ± 4.8; *P*-value = 0.0001). These findings also align with the conclusion of Stendell et al.'s Danish database analysis [10]. Further, age also showed an odd of 1 in our ANN, but neither the model's Pr(>|*z*|) nor the age (mean ± SD) of the failed group was found to differ statistically. A logistic regression study by Kim et al. also did not find age as a predictor [11]. Even the study by Tessler et al. evaluating the effect of age on the spinal anesthesia procedure hinted that age is a minor determinant of difficulty [12]. Although sex appeared as a determinant in the ANN (Pr(>|*z*|) value 0.031), univariate analysis showed a *P*-value of 0.84. Considering the overfitting tendency of ANN, the Pr(>|*z*|) value of 0.031 for sex was disregarded as a predictor. Further, none of the aforementioned studies analyzing the predictors found gender as a determinant for first-pass success, difficult, or failed spinal anesthesia except for the study by Prakash et al. [8]. They found the male gender to have an association with difficulty. The sex distribution among our failed and successful groups was similar.

Last but not least, the failure term is used in this study in the context of the failure of the procedure, not SAB. Failed SAB is usually reserved for those spinal blocks requiring general anesthesia, a second spinal procedure, or surgeon-infiltrated local anesthesia to proceed with the intended surgery [13].

This study has a few limitations as well. The power of the study was calculated for a composite outcome assessment, which might be inadequate for individual variables incorporated as input for ANN, especially for those parameters that just missed the significance level or attained an odds ratio (OR) of 1 or more; however, the 2.5% end of 95% confidence interval of the OR remained below 1. The number of obese and elderly patients was relatively lower; pregnant patients were included. Further, the gender distribution in the cohort had male predominance. Therefore, prospective studies with a larger sample, especially from multicenter design in the future might bring out other significant predictors.

## Conclusions

The present ANN model could identify the determinants of failed spinal arachnoid puncture, and the model showed a good AuROC and excellent specificity; sensitivity was, however, lower. The findings indicate that patients with an impalpable interspinous process and intervertebral space, having positioning difficulty, and procedures performed by performers with less than one year of experience are likely to require more punctures, passes, and procedural failure. Higher weight, BMI, and convex contour of the thoraco-lumbosacral spine are also likely to predict failed spinal arachnoid puncture procedures but with a weaker association. A multicenter study with a larger sample will provide more precise insights into these. 
